# Effect of the number of unhealthy lifestyles in middle-aged and elderly people on hypertension and the first occurrence of ischemic stroke after the disease

**DOI:** 10.3389/fcvm.2023.1152423

**Published:** 2023-05-30

**Authors:** Xiaopeng Zhu, Fang Zhang, Zhongyan Luo, He Liu, Xia Lai, Xiaotong Hu, Qian Xie, Xia Gao, Yan Long

**Affiliations:** ^1^ Department of Geriatric Medicine, Daping Hospital, Army Medical University (Third Military Medical University), Chongqing, China; ^2^ Department of Health Management, Daping Hospital, Army Medical University (Third Military Medical University), Chongqing, China; ^3^ Department of Family Medicine, Community Health Center of Daping Street, Yuzhong District, Chongqing, China

**Keywords:** hypertension, unhealthy lifestyle, ischemic stroke, middle-aged and elderly people, number of unhealthy lifestyles

## Abstract

**Background:**

To determine the relationship between the number of controllable unhealthy lifestyles on the risk of the first occurrence of ischemic stroke after the disease in middle-aged and elderly people in the community, and to provide data support and basis for community physicians to guide hypertensive patients to control modifiable risk factors to prevent the first occurrence of ischemic stroke.

**Methods:**

The relationship between the number of unhealthy lifestyles and the risk of hypertension was analyzed by binary logistic regression in 584 subjects using a medical record control study. A retrospective cohort study of 629 hypertensive patients was used to analyze the relationship between the number of unhealthy lifestyles and the risk of the first occurrence of ischemic stroke within 5 years of developing hypertensive disease using Cox proportional risk regression models.

**Results:**

Logistic regression model analysis showed that taking an unhealthy lifestyle as a reference, the OR (95% CI) values of, 2, 3, 4 and 5 unhealthy lifestyle were 4.050 (2.595–6.324), 4 (2.251–7.108), 9.297 (3.81–22.686), and 16.806 (4.388–64.365), respectively. Cox Proportional risk regression model analysis showed that the risk of ischemic stroke within 5 years after developing hypertension was referenced to 5 unhealthy lifestyles, and the HR (95% CI) for 3, 2, and 1 unhealthy lifestyle were 0.134 (0.023–0.793), 0.118 (0.025–0.564), and 0.046 (0.008–0.256), respectively.

**Conclusion:**

The number of controllable unhealthy lifestyles in middle-aged and elderly people was positively associated with the risk of hypertension and first ischemic stroke after hypertension, and there was a dose-effect relationship between them. The risk of hypertension and first ischemic stroke within 5 years after hypertension onset increased with the number of unhealthy lifestyles.

## 1. Introduction

More than 12.2 million new strokes occur each year worldwide (1), of which ischemic stroke is the most common type (2). Previous studies have shown many risk factors for ischemic strokes, such as hypertension, hyperlipidemia, and atrial fibrillation, as well as many modifiable risk factors, including lifestyle behaviors such as smoking, physical inactivity, and excessive alcohol consumption (3), but more than 60% of strokes, are caused by hypertension (4). According to Chinese guidelines (5), 244.5 million Chinese adults aged ≥18 years have hypertension and another 435.3 million have prehypertension. The high prevalence of hypertension and prehypertension in Chinese communities is associated with unhealthy lifestyles such as smoking, physical inactivity, and excessive alcohol consumption. One study confirmed that prevention of hypertension is crucial, while a 10 mmHg reduction in systolic blood pressure reduces the risk of stroke by approximately 1/3 (4). Another study showed that the best way to prevent disease in individuals is to change unhealthy lifestyles and that people who adopt a healthy lifestyle have an 80% lower risk of a first stroke than those who do not (6). Also, a study by the Global Burden of Disease (GBD) calls for effective primary prevention strategies to be implemented globally to reduce the incidence of stroke (1). However, most studies have been conducted on whether a controllable unhealthy lifestyle is a risk factor for hypertension and stroke (7–12), and few have reported on the quantitative impact of an unhealthy lifestyle on hypertension, and the number of unhealthy lifestyles may persist after hypertension, which has little impact on the risk of first ischemic stroke after hypertension. Relevant studies have been reported. Therefore, we investigated the relationship between the number of controllable unhealthy lifestyles and hypertension and its prognosis to encourage residents to control the number of unhealthy lifestyle actively preventon of hypertension and thus reduce the risk of ischemic stroke and the family burden.

## 2. Subjects and methods

### 2.1. Subjects

From 2019 to 2021, 292 middle-aged and older adults aged >45 years with hypertension and 292 controls each were recruited at Daping Community Hospital, and 629 hypertensive patients with complete follow-up information, all of whom were followed up by community physicians at least once a year by telephone, home visits, and outpatient visits. **Inclusion criteria:** (1). age >45 years; (2). able to cooperate with the completion of the follow-up in this study; (3). informed consent to the study and signed the informed consent form. **Exclusion criteria:** (1). history of previous ischemic stroke or hemorrhagic stroke with severe sequelae; (2). presence of other conditions causing hypertension and central nervous system, such as primary aldosteronism, metabolic encephalopathy, Parkinson's syndrome, Huntington's disease, subdural hematoma, normal cranial pressure hydrocephalus, brain tumor, traumatic brain injury, etc.; (3). presence of psychiatric disorders such as depression, schizophrenia, etc. (4). patients with severe heart, liver, kidney, and other vital organ diseases; (5). patients with visual impairment, aphasia, deafness, etc,. insufficient to perform follow-up; 6. patients who refused to sign the informed consent form.

This study adhered to the principles of the Declaration of Helsinki and was approved by the Ethics Committee of Daping Street Community Health Service Center, Yuzhong District, Chongqing, China (2019-01).

### 2.2. Methods

#### 2.2.1. General data collection

(1) demographic information: age, gender, education level, lifestyle, etc.; (2) medical history: the medical history of the study subjects was mainly obtained from medical records, including the history of previous trauma and surgery, psychiatric disease, coronary artery disease, and atrial fibrillation, chronic hepatitis, chronic renal insufficiency, coronary artery disease, diabetes mellitus, hyperlipidemia, Parkinson's disease, and history of related medications; (3) some Laboratory tests and ancillary findings: including three major routine tests, electrocardiogram, blood pressure, blood glucose, blood lipids, liver, and kidney function tests and cranial CT or MRI examination.

#### 2.2.2. Follow-up data collation

Community doctors followed up with the patients at least once a year by telephone, home visits, and outpatient visits, recording the general information of the patients and whether ischemic stroke occurred for the first time each year after having hypertension, and the hospital and time of diagnosis after the occurrence of ischemic stroke.

### 2.3. Observation variable

2.3.1. Smoking: as the average of at least 1 cigarette per day in the past 1 year, or quit smoking for less than 5 years (13).
2.3.2. Alcohol consumption as all daily consumption of liquor > 35 g/day (6).
2.3.3. Physical exercise: lack of exercise <3 times per week, each duration <30 min (2).
2.3.4 Diet: according to the weekday salt, high salt (>5 g/d) (14); sugary drinks >250 ml/day for sugar diet (15); oil diet ≥2 times/week: 2–6 times/week or 1–3 times/day (16).
2.3.5. Body mass index: when body mass index ≥ 24 kg/m2 was overweight (17, 18).

### 2.4. Follow-up and identification of outcome events

The first detection of hypertension in the observation subjects was used as the starting time of follow-up, which was defined as hypertension with blood pressure >130/80 mmHg according to the 2017 American College of Cardiology (ACC)/American Heart Association (AHA) guidelines for the management of hypertension (19). Stroke, death, or end of follow-up (2021/10/31) after 5 years of disease was used as the follow-up endpoint, and if the study subject died midway through the study without an outcome event, the time of death was used as the time of termination of follow-up. Ischemic stroke was diagnosed if signs and symptoms of focal neurological deficits were present in combination with imaging (cranial CT or magnetic resonance imaging) according to the criteria defined by the World Health Organization (20), and patients whose diagnosis was confirmed based on community physician records were identified.

### 2.5. Statistical methods

All data were analyzed using SPSS 26.0 software, and the Kolmogorov-Smirnov test for normality was applied. Count data were expressed as a number of cases (percentage), and non-normally distributed measure data were expressed as median (interquartile spacing). Wilcoxon Mann Whitney (rank-sum) test was used for non-normally distributed measure data in two subgroups. *χ*^2^ Spearman correlation analysis was used to correlate the number of the 5 unhealthy lifestyles with hypertension and the occurrence of the first ischemic stroke after the disease. Logistic regression models were used to analyze the relationship between the number of unhealthy lifestyles and hypertension; the Kaplan-Meier method was used to calculate the cumulative incidence of first ischemic stroke by the number of unhealthy lifestyles after having hypertension, and the Log-rank test was used for comparison between groups. The relationship between the number of unhealthy lifestyles after hypertension and the risk of first ischemic stroke was analyzed using Cox proportional risk regression models. All statistical plots were drawn using GraphPad Prism 9. Differences were considered statistically significant at *P* < 0.05.

## 3. Results

### 3.1. Basic information of the population with hypertension and control group

The present study included 292 middle-aged and elderly subjects with hypertension and their matched control group, and the basic information was shown in Table 1. There were no statistical differences in age, sex, history of coronary heart disease, history of diabetes, history of other chronic diseases, years of education, living environment, heart rate, and sleep between the 2 groups. Middle-aged and elderly patients with hypertensive disease had significantly higher rates of low annual personal income, rate of the father with the hypertensive disease, rate of the mother with hypertensive disease, BMI, lack of physical activity, rate of having dietary habits (oil, salt, sugar), rate of smoking, and rate of alcohol consumption than the control group (*P* < 0.05).

**Table 1:** Statistics of study population characteristics.

Basic information	CN (*n* = 292)	Hypertension (*n* = 292)	*χ*^2^ or *Z* value	*P-*value
Age, years	70.72 (66.00–76.00)	70.92 (66.00–76.75)	0.25	0.805
Sex (female) (%)	165 (56.50)	146 (50.00)	2.48	0.115
Years of education, years	9.00 (9.00–12.00)	9.00 (9.00–12.00)	2.76	0.431
Coronary heart disease (%)	60 (20.55)	55 (18.84)	0.27	0.603
Diabetes (%)	105 (35.96)	116 (39.73)	0.88	0.348
History of other chronic diseases (%)	11 (3.77)	15 (5.14)	0.64	0.422
Family history father (%)	31 (10.62)	48 (16.44)	4.23	0.040*
Family history mother (%)	41 (14.04)	72 (24.32)	10.55	0.001*
Income < 30,000yuan/year (%)	103 (35.27)	73 (25.00)	7.32	0.007*
Living environment (%)	3 (1.03)	8 (2.74)	2.32	0.128
Heart rate	77.00 (71.00–84.00)	76.00 (71.00–83.75)	0.52	0.607
BMI	24.30 (22.15–26.47)	25.80 (23.67–27.82)	19.15	<0.001*
BMI (<24 kg/m^2^) (%)	131 (44.86)	82 (28.09)		
Exercise (%)	102 (34.93)	218 (74.66)	93.02	<0.001*
Diet (%)	64 (21.92)	92 (31.51)	6.86	0.009*
Smoking (%)	61 (20.89)	97 (33.22)	11.25	0.001*
Drinking alcohol (%)	50 (17.12)	78 (26.71)	7.84	0.005*
Sleep (%)	88 (30.14)	103 (35.27)	1.75	0.186
5 unhealthy lifestyles (%)	3 (1.02%)	17 (5.82%)	8.66	0.002*
4 unhealthy lifestyles (%)	9 (3.08%)	27 (9.25%)	9.59	0.002*
3 unhealthy lifestyles (%)	38 (13.01%)	59 (20.21%)	5.45	0.02*
2 unhealthy lifestyles (%)	79 (27.05%)	136 (46.58%)	23.92	<0.001*
1 unhealthy lifestyles (%)	115 (39.38%)	53 (18.15%)	32.12	<0.001*
0 unhealthy lifestyles (%)	48(16.44%)	0(0%)	50.14	<0.001*

Table 1 Statistical information is expressed as the number of cases (percentages), and information on non-normally distributed measures is expressed as median (interquartile spacing). Wilcoxon Mann Whitney (rank-sum) test was used for non-normally distributed measures in both subgroups, and the *χ*^2^ test was used for the comparison of categorical variables.

*Indicates *P* < 0.05 compared with the control group.

### 3.2. Correlation between the number of unhealthy lifestyles and the risk of hypertension

**Figure 1:**
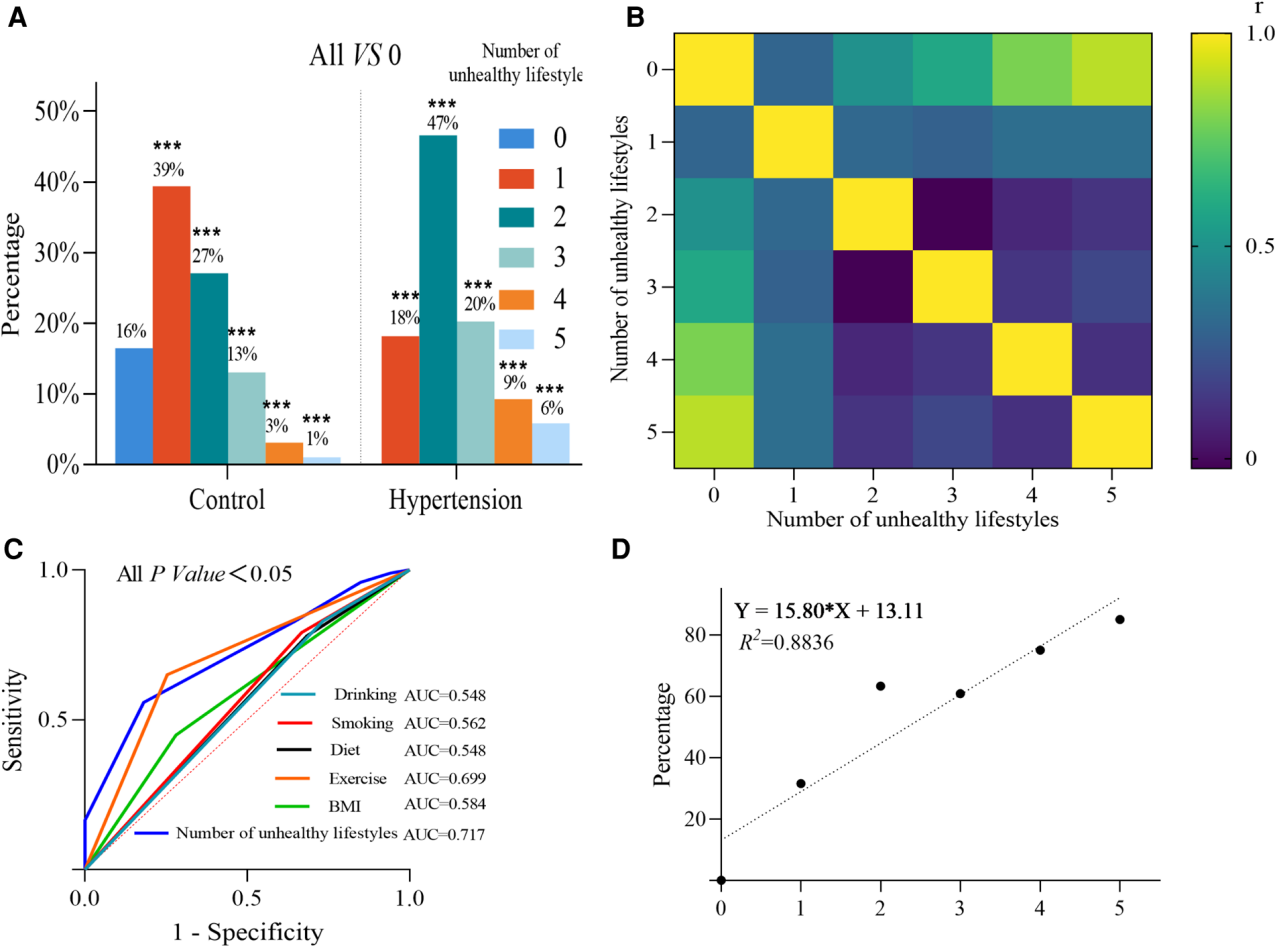
(**A**) Comparison of controls with different lifestyle counts for hypertension. (**B**) Correlation of hypertension with different lifestyle counts. (**C**) ROC curves for different lifestyles and counts. (**D**) Linear regression analysis of the rate of the ratio of those suffering from hypertension,with unhealthy lifestyle counts.

To illustrate the specificity of the correlation between the number of unhealthy lifestyles and the risk of hypertension, we first performed a chi-square test for the variability of the five unhealthy lifestyles in hypertension vs. controls. Compared with 0 unhealthy lifestyles, 1, 2, 3, 4, and 5 unhealthy lifestyles were statistically significant, with *P*-values < 0.001, and chi-square values of 20.067, 62.877 49.226, 53.053, and 54.400 (Figure 1A), indicating that the number of unhealthy lifestyles was significantly different in hypertensive patients and controls. Spearman correlation analysis was also performed between 0, 1, 2, 3, 4, and 5 unhealthy lifestyles. Compared with 0 unhealthy lifestyles, 1, 2, 3, 4, and 5 unhealthy lifestyles were statistically significant with *P*-values < 0.05, and the Correlation coefficient r were 0.305, 0.489, 0.583, 0.795 and 0.894, respectively (Figure 1B). To further clarify the correlation between the number of unhealthy lifestyles and the ratio of those suffering from hypertension, the linear regression analysis showed a strongly positive correlation with *P* < 0.05, R^2^ = 0.8836 (Figure 1D).

### 3.3. Number of unhealthy lifestyles and the risk of developing the hypertensive disease

**Figure 2:**
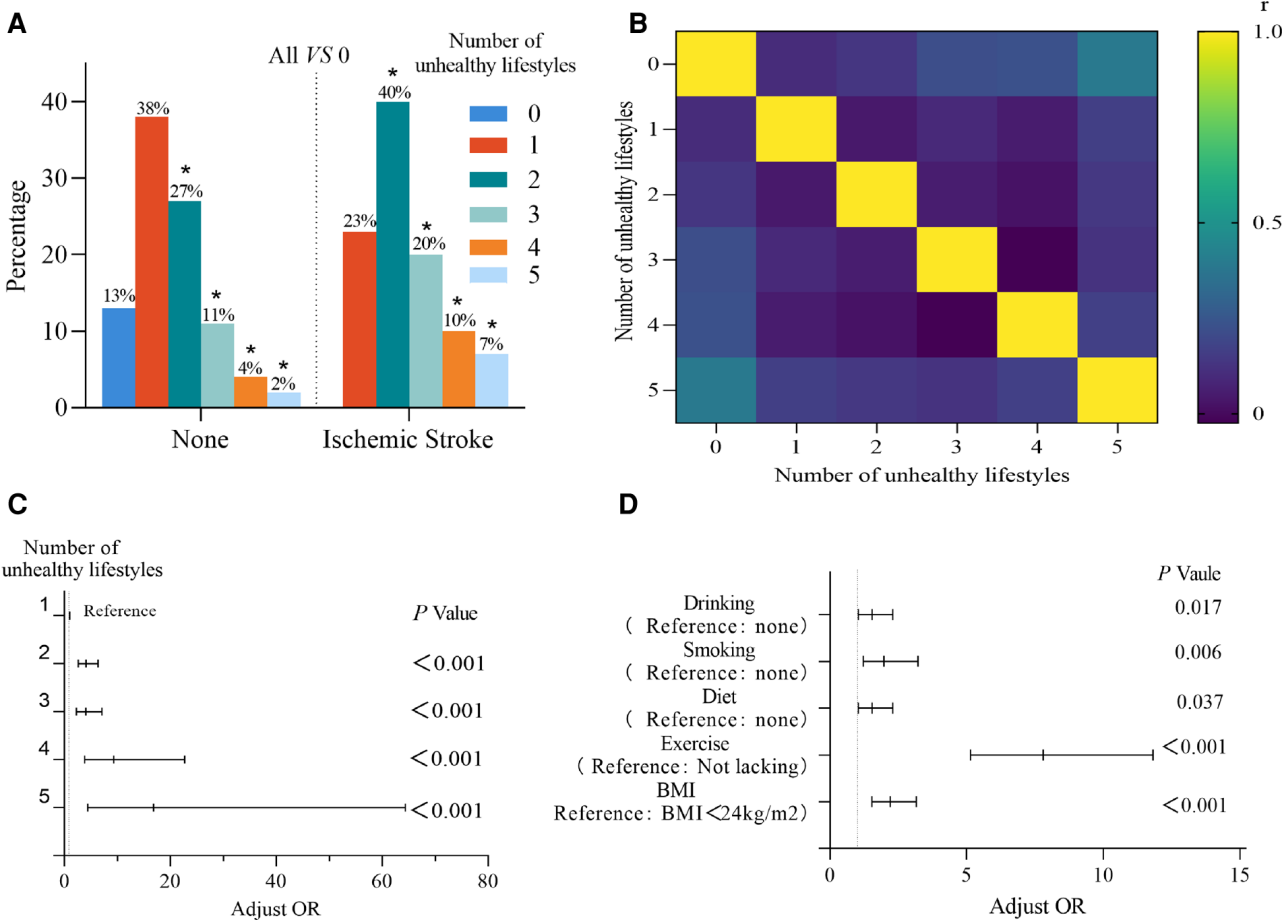
(**A**) Comparison of the number of ischemic strokes occurring in hypertension with the number of different lifestyles not occurring in hypertension. (**B**) Correlation between stroke and the number of unhealthy lifestyles. (**C**) Logistic regression analysis of the number of unhealthy lifestyles. (**D**) Logistic regression analysis of unhealthy lifestyles.

To test the reliability of previous literature reporting BMI, physical activity rate, diet, smoking, and alcohol consumption as independent risk factors for developing hypertension in our data, we corrected for age, history of coronary heart disease, history of diabetes mellitus, and history of other diseases, the binary logistic analysis showed the OR (95% CI) of BMI > 24 kg/m2, lack of exercise, having dietary preferences, smoking, and alcohol consumption were 2.205 (1.538-3.160), 7.798 (5.147-11.816), 1.55 (1.046-2.298), 1.98 (1.217-3.222), and 1.802 (1.112-2.919), as shown in the Figure 2D. To clarify the effect of the number of controllable unhealthy lifestyles on the disease, we corrected for age, history of coronary heart disease, history of diabetes, and history of other diseases by multifactorial binary logistic analysis. As shown in the Figure 2C, with having 1 unhealthy lifestyle as reference, the OR (95% CI) of 2, 3, 4, and 5 unhealthy lifestyles were 4.051 (2.595–6.324), 4.000 (2.251–7.108), 9.297 (3.81–22.686), and 16.806 (4.388–64.365), respectively (*p*<0.05). This indicates that as the number of unhealthy lifestyles increases, the risk of disease increases, with 2, 3, 4, and 5 being 4–16 times greater than 1 unhealthy lifestyle, respectively. To evaluate the accuracy of the number of unhealthy lifestyle on the prediction of hypertension risk, we plotted the ROC curve, and the area under the curve AUC (95% CI) of the number of unhealthy lifestyle was 0.717 (0.676–0.758), sensitivity was 81.8%, specificity was 55.8%, and Jorden index was 0.376, *P* < 0.001, the results were shown in Figure 1C. These results suggested the number of unhealthy lifestyle could predict the risk of hypertension.

### 3.4. Basic information on the number of unhealthy lifestyles and the population at risk of first ischemic stroke after developing hypertension

The present study included 629 middle-aged and elderly people with complete previous data on hypertension, 368 women (58.5%) with a mean age of (71.51 ± 0.366), 30 patients (4.77%) who had their first ischemic stroke after having hypertension, and 14 (2.23%) who had their first ischemic stroke within 5 years after having hypertension. The number of unhealthy lifestyles was shown in Table 2.

**Table 2:** Statistics of the number of unhealthy lifestyles

	**Number of unhealthy lifestyles**						
	0	1	2	3	4	5	Total
**Hypertension**(%)	84(13.35)	243(38.63)	184(29.25)	72(11.45)	29(4.61)	17(2.70)	629(100.00)
BMI(%)		132(54.32)	154(83.70)	61(84.72)	26(89.66)	17(100.00)	390(62.00)
Exercise(%)		68(27.98)	112(60.87)	44(61.11)	25(86.21)	17(100.00)	256(40.70)
Diets(%)		27(11.11)	56(30.43)	37(51.39)	19(65.52)	17(100.00)	156(24.80)
Drinking(%)		5(2.06)	23(12.50)	32(44.44)	23(79.31)	17(100.00)	100(15.90)
Smoking(%)		11(4.52)	32(17.39)	41(56.94)	23(79.31)	17(100.00)	124(19.71)
**Ischemic Stroke**(%)		7(23.33)	12(40.00)	6(20.00)	3(10.00)	2(6.67)	30(100.00)
BMI(%)		4(47.14)	8(66.67)	6(100.00)	3(100.00)	2(100.00)	23(76.67)
Exercise(%)		3(42.86)	5(41.67)	3(50.00)	1(33.33)	2(100.00)	14(46.67)
Diets(%)			4(33.33)	4(66.67)	2(67.67)	2(100.00)	12(40.00)
Smoking(%)			4(33.33)	4(66.67)	3(100.00)	2(100.00)	13(43.33)
Drinking(%)			3(25.00)	1(16.67)	3(100.00)	2(100.00)	9(30.00)

### 3.5. Correlation between the number of unhealthy lifestyles and the first occurrence of ischemic stroke after having hypertension disease

**Figure 3:**
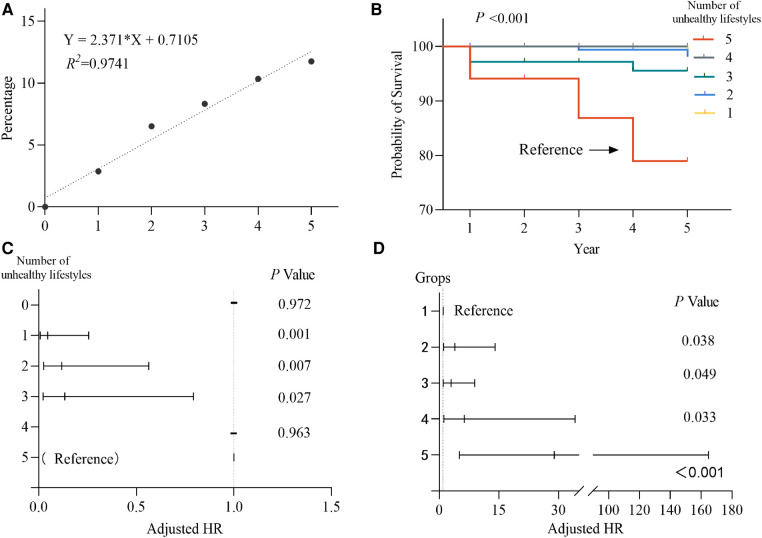
(**A**) Linear regression analysis of the number of unhealthy lifestyles on the incidence of stroke after hypertension. (**B**) Kaplan-Meier cumulative rate of ischemic stroke over 5 years. (**C**) Regression analysis of the proportion of COX by the number of unhealthy lifestyles. (**D**) Regression analysis of the proportion of COX by unhealthy lifestyle subgroups.

The persistence of an unhealthy lifestyle after developing hypertension may have an impact on the prognosis of hypertension, for which we statistically analyzed a total of 30 of 629 patients who had their first ischemic stroke after developing hypertension. We found that the first ischemic stroke after hypertension was statistically significant in patients with 2, 3, 4, and 5 unhealthy lifestyles compared to 0 unhealthy lifestyles (*P* < 0.05) with chi-square values of 5.735, 7.280, 8.927, and 10.082, respectively, while it was not statistically significant in patients with 1 unhealthy lifestyle (*P* = 0.116), as shown in the Figure 2A. Spearman correlation analysis was performed on 0, 1, 2, 3, 4 and 5 unhealthy lifestyles. Compared with 0 unhealthy lifestyles, the *P* value of 1 unhealthy lifestyle was 0.074, the *P* value of 2, 3, 4 and 5 unhealthy lifestyles were all less than 0.05, and the r value was 0.099, 0.133, 0.216, 0.228 and 0.389, respectively, as shown in Figure 2B. These results indicated a positive and weak association between the number of unhealthy lifestyles and the incidence of first stroke after hypertension. In order to further clarify the correlation between the number of unhealthy lifestyles and the prevalence rate of ischemic stroke occurring for the first time after hypertension, we analyzed the prevalence rates of ischemic stroke of 1, 2, 3, 4 and 5 unhealthy lifestyles: 2.88%, 6.52%, 8.33%, 10.34% and 11.76%, respectively. Linear regression analysis was shown in Figure 3A, and it was found that there was a strong positive correlation between the number of unhealthy lifestyles and the prevalence rate of ischemic stroke (R^2^ = 0.974, *P* < 0.05).

### 3.6. Number of unhealthy lifestyles and risk of first ischemic stroke after developing hypertension disease

We recorded that 14 of 629 hypertensive patients (2.23%) had their first ischemic stroke within 5 years. After determining the number of unhealthy lifestyles and the risk of first ischemic stroke within 5 years of hypertension as the equivalent risk, the Kaplan-Meier method was used to calculate the cumulative effect of the number of unhealthy lifestyles after having hypertension on the cumulative incidence of first ischemic stroke, and the results showed that there was a statistically significant difference (Figure 3B). And the result of Log-rank test showed that there was a statistically significant relationship between the five unhealthy lifestyles and the other quantities (*P* < 0.05), but no statistically significant relationship between the other quantities. Using a Cox proportional risk regression model to analyze the number of unhealthy lifestyles and the risk of first ischemic stroke after hypertension with 5 unhealthy lifestyles as a reference, no first ischemic stroke was observed in patients with 0 and 4 unhealthy lifestyles, and the HR (95% CI) for 3, 2, and 1 unhealthy lifestyle were 0.134 (0.023–0.793), 0.118 (0.025–0.564), 0.046 (0.008–0.256) and *P* < 0.05, as shown Figure 3C. With 0 and 1 unhealthy lifestyle as a reference, the first ischemic stroke in middle-aged and elderly hypertensive patients within 5 years at 2 or more, 3 or more, 4 or more, and 5 HR (95% CI) were 3.899 (1.081–14.062), 2.984 (1.003–8.876), 6.294 (1.161–34.117), and 28.897 (5.069–164.733), *P* < 0.05, as shown in the Figure 3D. It shows that the risk of first ischemic stroke in middle-aged and elderly hypertensive patients with 2 or more, 3 or more, 4 or more, and 5 were increased by 2.90, 1.98, 5.29, and 27.90 times, respectively, within 5 years, so the number of unhealthy lifestyles after having hypertensive disease significantly influences the occurrence of first ischemic stroke in patients.

## 4. Discussion

Non-pharmacological interventions are recommended for the majority of individuals in the prevention and treatment of hypertension, while unhealthy lifestyle changes are widely advocated as simple and easy for individuals to implement (21). Our findings suggest a dose-dependent relationship between the number of unhealthy lifestyles and the occurrence of hypertension and first ischemic stroke after hypertension, with an increased risk of developing hypertension and first ischemic stroke after hypertension with increasing numbers. To our knowledge, this is one of the few studies conducted to quantify the relationship between a controlled unhealthy lifestyle and hypertension and the first occurrence of ischemic stroke after developing hypertension.

Our analysis confirmed that the majority of hypertensive patients (more than 87%) had at least 1 of 5 controllable unhealthy lifestyles, which is the same as the previous conclusion that one of the 5 unhealthy lifestyles is a risk factor for developing hypertension (22). However, many previous studies limited the population, region, and race and cannot be fully generalized to our region (23–25), while the observational data from the Framingham study showed that individuals aged 55 to 65 years have a 90% lifetime risk of developing hypertension, so a study of the effect of the number of unhealthy lifestyles on hypertension in middle-aged and older adults in our region is very necessary (26). We found that of the five unhealthy lifestyles, lack of physical activity had the greatest risk of developing hypertension, followed by body mass index, with priority given to controlling physical activity over body mass index when the number of unhealthy styles was high or when residents were reluctant to control multiple, unlike a previous study that recommended priority control of body mass index (27). Another French study showed that three unhealthy lifestyle factors increased the odds of developing hypertension by 1.67 times compared to no unhealthy lifestyle (28), while we found the risk of developing hypertension was more than 16 times as high with five unhealthy lifestyles compared with one unhealthy lifestyle. These suggested dose effect between unhealthy lifestyles and hypertension. Our study included more unhealthy lifestyles than the former, and our criteria for blood pressure are more stringent than theirs proposed in the United States in 2017. Because the diet and drinking habits of the study population were also different, the implement ability for our community needs further study, so our conclusions are more meaningful for residents and patients in our community. Also, our analysis of ROC curves found that the number of different unhealthy lifestyles had a better judgment of the risk of developing hypertension than one unhealthy lifestyle, which proves that our analysis is more convincing and controllable selective, and can better guide doctors in the health management of hypertension for residents and patients.

We also found that 86% of patients still had at least one unhealthy lifestyle after developing hypertension, suggesting that controlling modifiable unhealthy lifestyles after developing hypertension is not ideal. A study by Katherine found that poor lifestyles (0–1 healthy behavior) led to an increased risk of all-cause mortality from stroke (29), and a US study showed that adult stroke patients were at higher risk for 4–5 poor lifestyle (30). A Chinese study showed increased mortality from ischemic stroke with the increasing number of 5 unhealthy lifestyles (31). These studies suggest that an increase in the number of unhealthy lifestyles may increase the risk of death or the risk of developing stroke. We found a strong dose-dependent relationship between the first occurrence of ischemic stroke after hypertension and the number of unhealthy lifestyles, with five unhealthy lifestyles being more than four times the incidence of one. We further investigated the effect of an unhealthy lifestyle on the occurrence of the first ischemic stroke within 5 years after hypertension, and the cumulative incidence of ischemic stroke within 5 years after hypertension was significantly different between 5 unhealthy lifestyles and other groups, whereas there was no statistically significant difference between 2, 3, and 4 groups, which may be since we did not observe ischemic stroke after hypertension for long enough, and possibly is the reason that the occurrence of ischemic stroke was not observed in patients with 0 and 4 types of unhealthy lifestyle hypertension that had an impact on the data analysis. For this reason, we performed group statistics, and the risk of more than 2 unhealthy lifestyles was 3 times higher than that of less than 1, and the risk of 5 unhealthy lifestyles was 28 times higher than that of less than 1. This may be the result of a synergistic effect between unhealthy lifestyles, so we recommend that patients with hypertension should control at least 2 unhealthy lifestyles to reduce the occurrence of ischemic strokes. Lacunar infarction is one of the most common subtypes of ischemic stroke and is often overlooked for prevention because it is a small-vessel lesion of the brain, so a manageable number of poor lifestyle changes may be able to benefit from it (32). Also, gender, grade of blood pressure, and increasing age may be independent risk factors for the occurrence of ischemic stroke, for this reason, the results remain after stratification by age, grade of blood pressure, and gender, and these potential biases do not necessarily affect the relationship between the number of unhealthy lifestyles we observed and the occurrence of first ischemic stroke after having the hypertensive disease.

Limitations of the present study: the occurrence of ischemic stroke is likely to be influenced by unhealthy lifestyles in a long and cumulative process, and the limited time and area of our observations allowed us to use only the incidence of the disease, not the prevalence, which may have biased the results somewhat. The specific values of their lipids were not recorded in the observation subjects, but only in other chronic diseases. Also, some patients' lipids returned to normal after taking lipid-lowering drugs, however, the damage to blood vessels from previous high lipid levels became a residual confounding factor, which may be another important risk factor for the occurrence of ischemic stroke. The number of unhealthy lifestyles may be reduced by being unaware of the risk factors themselves or not being diagnosed at the time of the unhealthy lifestyle assessment of the observed subjects. There may also be other factors that may influence disease progressions, such as prodromal diabetes, insulin resistance, unhealthy nutrition, or the use of aspirin, statins, angiotensin-converting enzyme inhibitors, angiotensin II receptor blockers (33–35). These factors may cause some bias in the results. There may also be potential effects of medication for other diseases and socioeconomic factors (personal economy, education). Thus our results may be limited by data, bias, unmeasured confounders, and residual confounders.

In conclusion, we found a strong positive association between the number of unhealthy lifestyles and hypertension and the first occurrence of ischemic stroke after the disease, and the causal relationship between an increase in the number of unhealthy lifestyles leading to an increased risk of developing hypertension and an increased risk of ischemic stroke after developing hypertension is clear. We recommend trying to control all unhealthy lifestyles, and if not, then increasing physical activity first, followed by weight control and smoking, and finally drink and diet,which may provide guidance to physicians in the management of hypertension and patients themselves in controlling the number of unhealthy lifestyles that can be modified to prevent ischemic stroke. This provides essential substantial evidence for future physicians in guiding patients in the management of hypertension and patients' control of unhealthy lifestyle changes to prevent the first occurrence of ischemic stroke.

## Data Availability

The original contributions presented in the study are included in the article/Supplementary Material, further inquiries can be directed to the corresponding author.
